# Supplementary material for: Development of a computer-aided design software for the quantitative evaluation of aesthetic damage
Source: PLoS One. 2019 Dec 18;14(12):e0226322. doi: 10.1371/journal.pone.0226322 (PMC6919621; doi:10.1371/journal.pone.0226322)
Supplement: S1 Code site — 1. (ZIP) [file pone.0226322.s001.zip › [EESC_jr][Projeto]Relatorio_07_21/reports/templates_zip/gentelella-master/vendors/morris.js/examples/weeks.html]

# Formatting Dates With Weeks

```
var week_data = [
  {"period": "2011 W27", "licensed": 3407, "sorned": 660},
  {"period": "2011 W26", "licensed": 3351, "sorned": 629},
  {"period": "2011 W25", "licensed": 3269, "sorned": 618},
  {"period": "2011 W24", "licensed": 3246, "sorned": 661},
  {"period": "2011 W23", "licensed": 3257, "sorned": 667},
  {"period": "2011 W22", "licensed": 3248, "sorned": 627},
  {"period": "2011 W21", "licensed": 3171, "sorned": 660},
  {"period": "2011 W20", "licensed": 3171, "sorned": 676},
  {"period": "2011 W19", "licensed": 3201, "sorned": 656},
  {"period": "2011 W18", "licensed": 3215, "sorned": 622},
  {"period": "2011 W17", "licensed": 3148, "sorned": 632},
  {"period": "2011 W16", "licensed": 3155, "sorned": 681},
  {"period": "2011 W15", "licensed": 3190, "sorned": 667},
  {"period": "2011 W14", "licensed": 3226, "sorned": 620},
  {"period": "2011 W13", "licensed": 3245, "sorned": null},
  {"period": "2011 W12", "licensed": 3289, "sorned": null},
  {"period": "2011 W11", "licensed": 3263, "sorned": null},
  {"period": "2011 W10", "licensed": 3189, "sorned": null},
  {"period": "2011 W09", "licensed": 3079, "sorned": null},
  {"period": "2011 W08", "licensed": 3085, "sorned": null},
  {"period": "2011 W07", "licensed": 3055, "sorned": null},
  {"period": "2011 W06", "licensed": 3063, "sorned": null},
  {"period": "2011 W05", "licensed": 2943, "sorned": null},
  {"period": "2011 W04", "licensed": 2806, "sorned": null},
  {"period": "2011 W03", "licensed": 2674, "sorned": null},
  {"period": "2011 W02", "licensed": 1702, "sorned": null},
  {"period": "2011 W01", "licensed": 1732, "sorned": null}
];
Morris.Line({
  element: 'graph',
  data: week_data,
  xkey: 'period',
  ykeys: ['licensed', 'sorned'],
  labels: ['Licensed', 'SORN']
});
```
